# Study of the Chemical Composition of the Resinous Exudate Isolated from *Heliotropium Sclerocarpum* and Evaluation of the Antioxidant Properties of the Phenolic Compounds and the Resin

**DOI:** 10.3390/molecules14114625

**Published:** 2009-11-12

**Authors:** Brenda Modak, Melissa Salina, Jesús Rodilla, René Torres

**Affiliations:** 1 Departamento de Ciencias del Ambiente, Facultad de Química y Biología, Universidad de Santiago de Chile, Santiago, Chile; E-Mails: salinas.meli@gmail.com (M.S.); rene.torres@usach.cl (R.T.); 2 U. I&D Materiais Textêis e do Papel, Departamento de Química, Universidade da Beira Interior,6201-001 Covilhã, Portugal; E-Mail: rodilla@ubi.pt (J.R.)

**Keywords:** *Heliotropium sclerocarpum*, flavonoids, 2-arylbenzofuran, geranyl derivative, antioxidant capacity, resinous exudate

## Abstract

*Heliotropium sclerocarpum* Phil. (*Heliotropiaceae*) is a resinous bush that grows in the Atacama of northern Chile. The chemical composition of its resinous exudate was analyzed for the first time. One aromatic geranyl derivative: filifolinol (**1**), one flavanone: naringenin (**2**) and a new type of 3-oxo-2-arylbenzofuran derivative **3** were isolated and their structures were determined. The antioxidant activity of the phenolic compounds and resin was evaluated using the bleaching of DPPH radical method and expressed as fast reacting equivalents (FRE) and total reacting equivalents (TRE).

## 1. Introduction

Plants that grow in desert zones are characterized by producing a resinous exudate from their glandular trichomes that covers the surface of leaves and stems. This exudate has been described as a defense mechanism against extreme conditions of the environment [1]. In general, the most common and structurally diverse compound group found in glandular trichomes exudates are terpenoids, together to phenylpropanoids (flavonoids and allied phenolics). Flavonoids occur as aglycones in the trichome exudates of several species, sometimes as crystalline deposits. The occurrence of flavonoids as aglycones is not surprising since they are lipophillic and would be highly soluble in mixed exudates containing terpenoids [2].

In order to elucidate the role of secondary metabolites isolated from resinous exudates in the defense of native plants that growing in arid zones of Chile exposed to extreme conditions, particularly species of the *Heliotropium* genus, we have been performing phytochemical analyses of these exudates. The results have shown that the resin was characterized by the presence of flavonoids as main components, complemented with aromatic geranyl derivatives [3,4,5,6,7,8,9,10,11]. The high concentration of flavonoids is associated with a protective role against the oxidative stress imposed on the species and their well known antioxidant capacity [12].

We now report the results of our chemical study of the resinous exudate of *Heliotropium sclerocarpum* Phil., family *Heliotropiaceae*. This species is an endemic resinous bush that grows in Huasco some 58 kms northwest of Vallenar (III region, Chile). The antioxidant activity of the isolated phenolic compounds and of the resin was also measured using the DPPH radical [(1,1-diphenyl-2-picrylhydrazyl)] bleaching method. The results were expressed in microequivalents of Trolox^®^ through the determination of its fast (FRE) and total (TRE) reacting equivalents [1], where FRE is a good measure of the quick protection of a given compound against oxidants and TRE measures the degree of long-term protection of the antioxidant, or how effective it is against a strong oxidative stress.

## 2. Results and Discussion

### 2.1. Analysis of the chemical composition of the resin

**Figure 1:**
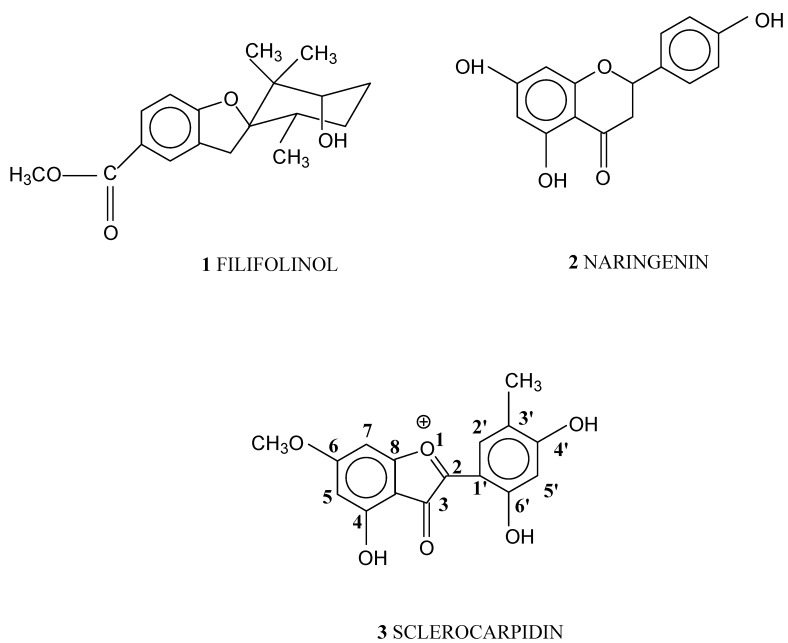
Compounds isolated from resinous exudates obtained of *Heliotropium sclerocarpum* Phil.

The chemical composition of the resinous exudate produced by *Heliotropium sclerocarpum* Phil. was similar to those found in other species of *Heliotropium* genus, characterized by the presence of aromatic geranyl derivatives and flavonoids. From the resin of *Heliotropium sclerocarpum* Phil. three main secondary metabolites were isolated: one aromatic geranyl derivative, namely filifolinol (**1**), the flavanone naringenin (**2**) and 2-(4’,6’-dihydroxy-3’-methylphenyl)-4-hydroxy-6-methoxy-3-oxo-benzofurylium (**3**), a new type of 3-oxo-2-arylbenzofuran derivative that we have named sclerocarpidin (Figure 1). Filifolinol had been previously obtained from *Heliotropium filifolium* and *H. taltalense* [4,11] and naringenin had been found previously in the resinous exudates of *H. stenophyllum*, *H. sinuatum*, *H. chenopodiaceum* and *H. taltalense* [3,4,5,11]. Their structures were confirmed by comparison with authentic samples and with literature spectral data. The new compound **3** was characterized by its spectroscopic data. Compound **1** was obtained as yellow crystals. The UV spectra recorded using MeOH as solvent showed similar signals to bands I and II of anthocyanidins and arylbenzofurans (see Experimental). Bathocromic shifts upon addition of NaOAc indicated the presence of hydroxyl group at C-4’, characteristic of the following chromophore:

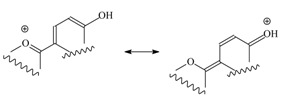


This compound was red colored when dissolved in an acid medium (HCl) and changed to a blue color when dissolved in a basic medium, confirming the presence of 3-oxo-2-arylbenzofuran derivative. The presence of hydroxyl and carbonyl groups was evident from the IR absorption bands at 3,350 cm^-1^ and 1,700 cm^-1^ respectively. Also, the signal at 1,628 cm^-1^ showed aromatic C=C stretching. The ^1^H-NMR spectrum showed two signals (s, 1H) at δ 12.11 and δ 12.31 ppm corresponding to two phenolic hydroxyl groups which appear to low field because they form hydrogen bonds with the carbonyl group. These signals were assigned to OHs attached to C-4 and C-6’ respectively. A signal at δ 2.45 ppm (s, 3H, CH_3_- C-3’) could be attributed to a methyl group located on ring B. A DEPT experiment indicated the presence of four CH aromatics and one methyl group attached to an aromatic carbon. The proton at δ 2.45 ppm showed relation with the carbon at δ 22.12 ppm in the HSQC experiment. This signal also showed a long-distance connection with the carbons at 121.25 ppm (C-5’) and 124.47 ppm (C-2’) in the HMBC experiment. The signal at δ 3.93 ppm (s, 3H, OCH_3_) is characteristic of a methoxyl group. These protons were related with the ^13^C-NMR signal at δ 56.04 ppm in the HSQC experiment. Four signals were obtained in the aromatic area at δ 6.68 and δ 7.37 ppm (two doublets, 1H, *J* = 2.56 Hz, H-5 and d, 1H, *J* = 2.56 Hz, H-7, respectively). Both signals were shown to be *meta* coupled by a COSY experiment and to be related with the ^13^C-NMR signals at δ 106.72 and δ108.19 ppm, respectively, in the HSQC experiment. These signals are characteristic of the *meta* protons of the ring A of flavonoids. Two singlets at δ 7.08 and δ 7.63 ppm (s, 1H, H-2’ and s, 1H, H-5’, respectively) were attributed to two aromatic protons in the ring B in the only positions available. These four aromatics protons had been indicated already by DEPT experiment. The protons at δ 7.08 and δ 7.63 ppm were related with the ^13^C-NMR signals at δ 124.47 and δ121.25 ppm, respectively, in the HSQC experiment. The ^13^C-NMR spectrum presented signals for 16 carbons of which four are CH aromatics, one methyl group united to aromatic ring, one methoxyl group and a carbonyl group. The others are carbons with substituents. The assigned structure was confirmed by the high resolution M+Na ion at m/z 323.1410 (calcd. by C_16_H_13_O_6_Na). This rare structure was identified in the nature for the first time.

### 2.2. Antioxidant activity

The antioxidant activity of the phenolic compounds **2** and **3** and of the resin was evaluated using the bleaching of DPPH radical method and expressed in terms of FRE (fast reacting equivalents) and TRE (total reacting equivalents). FRE is a measure of the quick protection of a given compound against oxidants and TRE measures the degree of long-term protection of the antioxidant [10,12]. The results of the assay are summarized in Table 1. The radical scavenging activity displayed by the extract in this assay would thus confirm the protective role of the resin towards oxidant species present in the environment. The values of FRE and TRE are superior in comparison with those obtained for the pure compounds. This indicates that the resin offers to the plant instantaneous protection and also in the long term. On the other hand, is known that the structure of the phenolic compounds are adapted to capturing free radicals due to the ease with which the hydrogen of the hydroxyl group can be donated to the radical and to generate a more stable structure. Therefore, the number and localization of the hydroxyl groups would be clearly involved in the antioxidant activity of these compounds [13,14,15,16]. The compound **3**, with three hydroxyl groups, showed very low activity values, both for FRE as for TRE. This result can be interpreted in two ways. First, is possible that the hydroxyl groups in position 4 and 6’ do not participate in the antioxidant activity because they are forming hydrogen bonds with the carbonyl group. Therefore, the only free hydroxyl group is the one attached to C-4’ in the B ring. Second, this reflects the fact that the reaction is very slow and is not complete in 15 minutes. In this respect naringenin (**2**), which also has three hydroxyl groups, showed significantly higher values. For this compound, the value of TRE is much greater than the FRE, indicating that mainly it offers the plant long-term protection. On the other hand, naringenin (**2**) has been reported to protect human cells from oxidative stress [17,18]. The antioxidant effect produced by a given compound will be determined by its concentration and reactivity [19]. In this way, the high concentration of **2** found in *H. sclerocarpum* resin, in relation to other pure compounds, must be directly related to a mechanism of protection of the plant cells against oxidative damage in the resin. The antioxidant effect of a complex mixture, such as resinous exudates, will depend of the quantity and quality of the antioxidants present [19]. In this case, the antioxidant activity of the total extract is higher than the isolated compounds. This can be the result of some form of synergism of their components.

**Table 1:** Experimentally determined indexes of FRE and TRE for the reaction of the DPPH radical with the isolated compounds **2** and **3** and the resinous exudates from *Heliotropium sclerocarpum*.

Compounds	Number of phenolic groups	FRE	TRE
**2**	3	7 × 10^-3^	3.6 × 10 ^-2^
**3**	3	7 × 10^-4^	8.7 × 10^-4^
Resin	-	5.9 × 10^-2^	18.2 × 10^-2^
Trolox^®^	1	1.0	1.0

On the other hand, the percentage of DPPH radical consumed by *H. sclerocarpum* was also determined and compared with the values of other resins obtained from *Heliotropium* species under identical conditions. These results are shown in Table 2. The level of DPPH radical consumed by the total extract of *H. sclerocarpum* is similar to those of resinous exudates isolated from other *Heliotropium* species at equal concentration and reaction time. Although the total consumption of DPPH radical is not observed, if it is significant. This demonstrates that these resins must play a protective role against the oxidative stress imposed by the environmental conditions in which they are developed.

**Table 2:** Comparison of the percentage of DPPH radical consumed by the resin obtained of *H. sclerocarpum*, *H. filifolium*, *H.sinuatum* and *H. stenophyllum.*

Resinous exudate	% DPPH consumed
Resin of *Heliotropium sclerocarpum*	47.3
Resin of *Heliotropium filifolium*	44.3
Resin of *Heliotropium sinuatum*	48.9
Resin of *Heliotropium stenophyllum*	20.2

According to the bibliography, the DPPH stable free radical method is an easy, rapid and sensitive way to survey the antioxidant activity of a specific compound or plant extracts [20]. Most of the studies in the literature are focused on the use of this method to only determine the reactivity of compounds or extracts in the search of potential antioxidant sources [21]. Nevertheless, we have given a different approach, aiming at the different type of protection that the compounds and the resinous exudate give to the plants according to the environmental conditions in which they are developed.

These results are in agreement with the literature, which propose that the production of plant phenolic compounds are thought to be part of the chemical defense of the plant, is under the control of both genetic and environmental factors [22].

## 3. Experimental

### 3.1. General

^1^H- (400 MHz) and ^13^C-NMR spectra (100 MHz) were recorded in CDCl3 on a Bruker Avance DRX400 spectrometer with TMS as internal standard. IR spectra were recorded on a Perkin-Elmer 735-B spectrophotometer. UV absorbance was measured using a Shimadzu UV-160 spectro-photometer. Mass spectra were obtained with a Fisons Autospec-Q VG-Analytical instrument. The optical rotations were measured with a Perkin-Elmer 241 polarimeter. The melting points were measured on a Kofler micro melting instrument and are not corrected. Known compounds were identified by comparison of their spectroscopic data with those in the literature and by co-chromatography with authentic samples. Silica gel 60 (70-230 mesh ASTM; 63-200 μm) for open column chromatography (CC) and GF254 for analytical TLC were purchased from Merck Ltd. (Germany). DPPH was purchased from Sigma-Aldrich Chemical Co. (USA). All solvents and chemicals used were of analytical grade. Standards of filifolinol and naringenin were obtained from Laboratory of Natural Products Chemistry of the Faculty of Chemistry and Biology of the University of Santiago of Chile.

### 3.2. Plant material

*Heliotropium sclerocarpum* Phil. was collected in October 2005 from Huasco, 3rd Region of Atacama, Chile (28º27` S, 71º13`W). A voucher specimen (HG 2907) was deposited at the Herbarium of Natural History Museum, Santiago of Chile.

### 3.3. Isolation and characterization of constituent of the resin

The fresh plant (960 g) was dipped into dichloromethane (500 mL) for 30 s. The organic extract was concentrated in a rotatory evaporator to give a residue of 15.65 g of resin. Seven g of the resinous exudate were separated into 27 fractions by CC (silica gel, mixtures of increasing polarity of chloroform-methanol as eluents). Fraction 17 (100 mg) was purified by PTLC on silica gel, eluting with hexane-ethyl acetate (95:5) to give 23.0 mg of 2-(4’,6’-dihydroxy-3’-methylphenyl)-4-hydroxy-6-methoxy-3-oxo-benzofurylium. [α]_D_^24°^ = -18.02° (*c* 1.9, CH3OH); mp: 199-202 ºC; IR (KBr) ν _max_ 3350, 2922, 1700, 1628 cm^-1^. UV (MeOH) λ_max_ 430, 290 (sh), 250 (sh), 230. UV (MeOH + NaOAc) λ_max_ 520, 302 (sh), 270. ^1^H-NMR: δ 2.45 (s, 3H, CH_3_), 3.93 (s, 3H, OCH_3_), 6.68 (d,1H, *J* = 2.56 Hz, H-5), 7.08 (s, 1H, H-2’), 7.37 (d, 1H, *J* = 2.56 Hz, H-7), 7.63 ( s, 1H, H-5’), 12.11 ( s, 1H, OH-C-4), 12.31 ( s, 1H, OH-C-6’). ^13^C-NMR: δ 22.12 (CH_3_), 56.04 (OCH_3_), 106.72 (C-5), 108.19 (C-7), 110.2 (C- 3’), 113.63 (C- 9), 121.25 (C-5’), 124.47 (C-2’), 133.16 (C-8), 135.2 (C-1’) 148.40 (C-4), 162.45 (C-4’) , 165.14 (C-6’), 165.50 (C-6), 182.0 (C-2), 190.76 (C-3). HRMS :M+Na ion *m*/*z* 323.1410.

Filifolinol **1** (17.0 mg) was previously obtained from *Heliotropium filifolium* and *H.taltalense* [4,11]. The flavonoid naringenin **2** (32.8 mg) had been found previously in the resinous exudates of *H. stenophyllum*, *H. sinuatum*, *H. chenopodiaceum* and *H. taltalense* [3,4,5,11].

### 3.4. Antioxidant activity determination

Solutions of antioxidant were prepared in ethanol (1 mg/mL). Aliquots of those solutions (100 μL) were added to the ethanolic radical solution (3 mL, 75 μM). Changes in the absorbance of the solution elicited by addition of the solutions containing the antioxidants were measured at 517 nm as a function of the elapsed time [19]. The antioxidant potential in the resinous exudates was obtained by monitoring their capacity to bleach DPPH radical. The equivalent antioxidant potential was obtained, in terms of Trolox^®^ equivalents, with the following formulas [23]:

(FRE)_Phenol_ = [(ΔºA)_phenol_/ ΔºA)_Trolox_)] (Trolox)/(Phenol)

(FRE)_resin_ = [(ΔºA)_resin_(ΔºA)_Trolox_] (Trolox)

(TRE)_Phenol_ = [Δ^15^A))_phenol_/ Δ^15^A)_Trolox_] (Trolox)/(Phenol)

(TRE)_resin_ = [(Δ^15^A))_resin_/ (Δ^15^A)_Trolox_] (Trolox)

where ΔºA is the decrease in absorbance (517 nm) measured 10 sec after the scavenger addition. Δ^15^ is the decrease in absorbance (517 nm) measured after 15 minutes addition.

The incorporation of additives (either the pure antioxidant compounds or the resinous exudates) produces a decrease in absorbance that is directly related to their capacity to bleach the radical. For most compounds, the bleaching does not occur instantaneously, but is generally complete after 15 minutes reaction. We have evaluated the "prompt" (measured ca 10 sec after addition) and total bleaching (measured after 15 minutes). A comparison of these values with that obtained instantaneously after Trolox (1 mM) addition provides an estimation of the total amount of compound that react instantaneously (FRE) and that react slowly with the pre-formed radical (TRE).

The percentage of DPPH radical consumed was obtained as:

% DPPH consumed = [A_0_ – A_15_/A_0_] × 100

where A_0_ is the absorbance measured 10 sec after the resin addition. Δ^15^ is the absorbance measured after 15 minutes addition.

## 4. Conclusions

From the resinous extract of *Heliotropium sclerocarpum* Phil. a new type of 3-oxo-2-arylbenzofuran derivative **3** was identified as 2-(4’,6’-dihydroxy-3’-methylphenyl)-4-hydroxy-6-methoxy-3-oxo-benzofurylium together with two other compounds: filifolinol (**1**), isolated previously from *H. filifolium* and *H. taltalense* and the flavonoid naringenin (**2**), identified previously in the resinous exudates of *H. stenophyllum*, *H. sinuatum*, *H. chenopodiaceum* and *H. taltalense*. This is the first time that this type of structure is obtained from a *Heliotropium* species. Also, **3** is a new compound in Nature. The antioxidant activity of the phenolic compounds and the extract was evaluated. Other species of the genus *Heliotropium* have been shown to produce resinous exudates with high concentrations of phenolic compounds, mainly flavonoids, in order to prevent the oxidative degradation of the resin that protects the plant [3,4,5,6,7,8,9,10,11]. In *H. sclerocarpum* the antioxidant activity was due mainly to naringenin.
